# LASER Assisted Excision of Solitary Neurofibroma in the Gingiva

**DOI:** 10.7759/cureus.7118

**Published:** 2020-02-27

**Authors:** Deepak M Ravindran, Shravanthy Ravi, Muthukumar Santhanakrishnan, Balaji SK

**Affiliations:** 1 Periodontology, Sri Ramachandra Institute of Higher Education and Research, Chennai, IND

**Keywords:** neurofibromatosis, gingival enlargement, laser

## Abstract

Neurofibromatosis (NF) is a genetic disorder that presents as benign tumours of the nervous system originating from the nerve sheath. It is of three types: Type I, Type II and Schwannomatosis. Type I Neurofibromatosis or von Recklinghausen's disease is the most common type of neurofibromatosis seen and it accounts for 90% of all cases seen. It can be seen as light brown pigmentation spots (Café-au-lait) on the skin and multiple small tumours among the nerves. Oral manifestations of NF are very rare and can be seen as sessile lesion in the tongue or the gingiva. The major complaints of the oral manifestations include difficulty in speech and mastication which it results in progression of periodontal disease. Here, we present a case report of Type I NF which presented as a sessile lesion in the right maxillary gingiva, for which we performed an excisional biopsy using LASER.

## Introduction

Gingival enlargement has many causes and can manifest with various characteristics. It can be transient or irreversible in nature [1].While the most common form of gingival enlargements results from inflammation and the usage of medications, it can also be indirectly associated with changes in the dimensions of underlying osseous tissues and other neoplastic lesions which manifest as fibrotic mass. Studies have reported that the presence of gingival enlargement associated with other systemic diseases will jeopardize the oral health status of the patient. In addition to systemic diseases, genetic disorders like Neurofibromatosis have also been reported to have oral manifestations in 72% of the affected individuals [2].

Neurofibromatosis (NF) is an autosomal dominant disorder that manifests as benign tumours of the peripheral nerve sheath in the sympathetic, peripheral or cranial nerves characterized by proliferating Schwann cells, perineural cells and fibroblasts of the endoneural origin [3]. Neurofibromatosis is of three types: Type I Neurofibromatosis (NF1) or von Recklinghausen's disease, Type II Neurofibromatosis (NF2) and Schwannomatosis. While Type I Neurofibromatosis is the most prevalent form (accounts for 90% of all cases seen), Type II and III are not seen frequently [4]. NF1 occurs due to alterations in the NF1 gene which is present on the long arm of chromosome 17 (17q11.2) [5]. Early detection of oral manifestation of neurofibromatosis does not happen frequently as these lesions are more symptomatic and are readily diagnosed when the patient complains of a mass or discomfort during speech or mastication. Here, we report a case of NF1 which manifested as a sessile lesion in maxillary right gingiva and was excised using LASER.

## Case presentation

**Figure 1:**
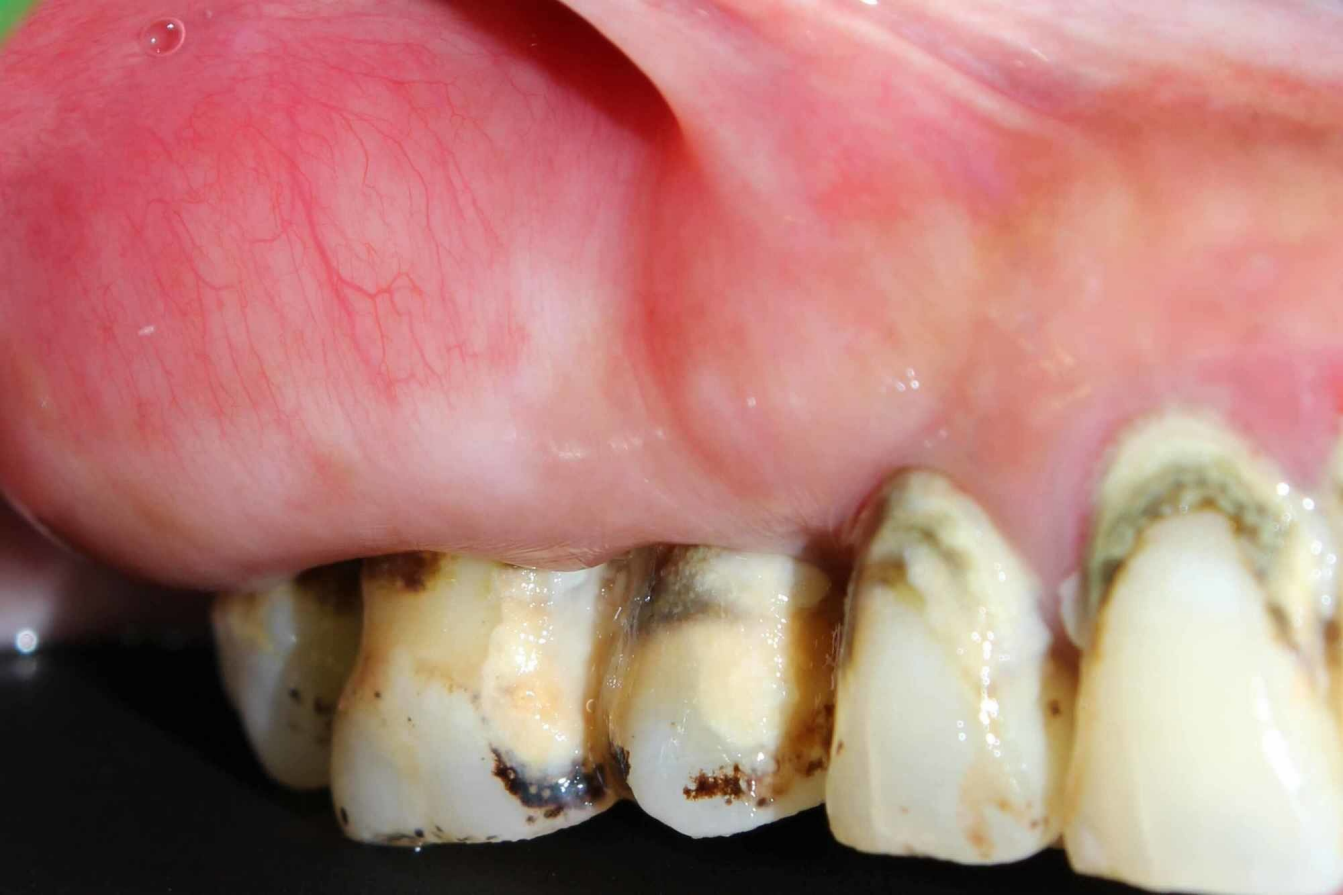
Pre - Operative Image of the Lesion extending from #14 to #17

A 42 year old female patient was referred to the Department of Periodontology, Faculty of Dental Sciences, Sri Ramachandra Institute of Higher Education and Research (SRIHER) for the excision of a painless swelling present in the maxillary right gingiva which was interfering with the denture given for replacement of maxillary anterior teeth [Fig 1].

**Figure 2:**
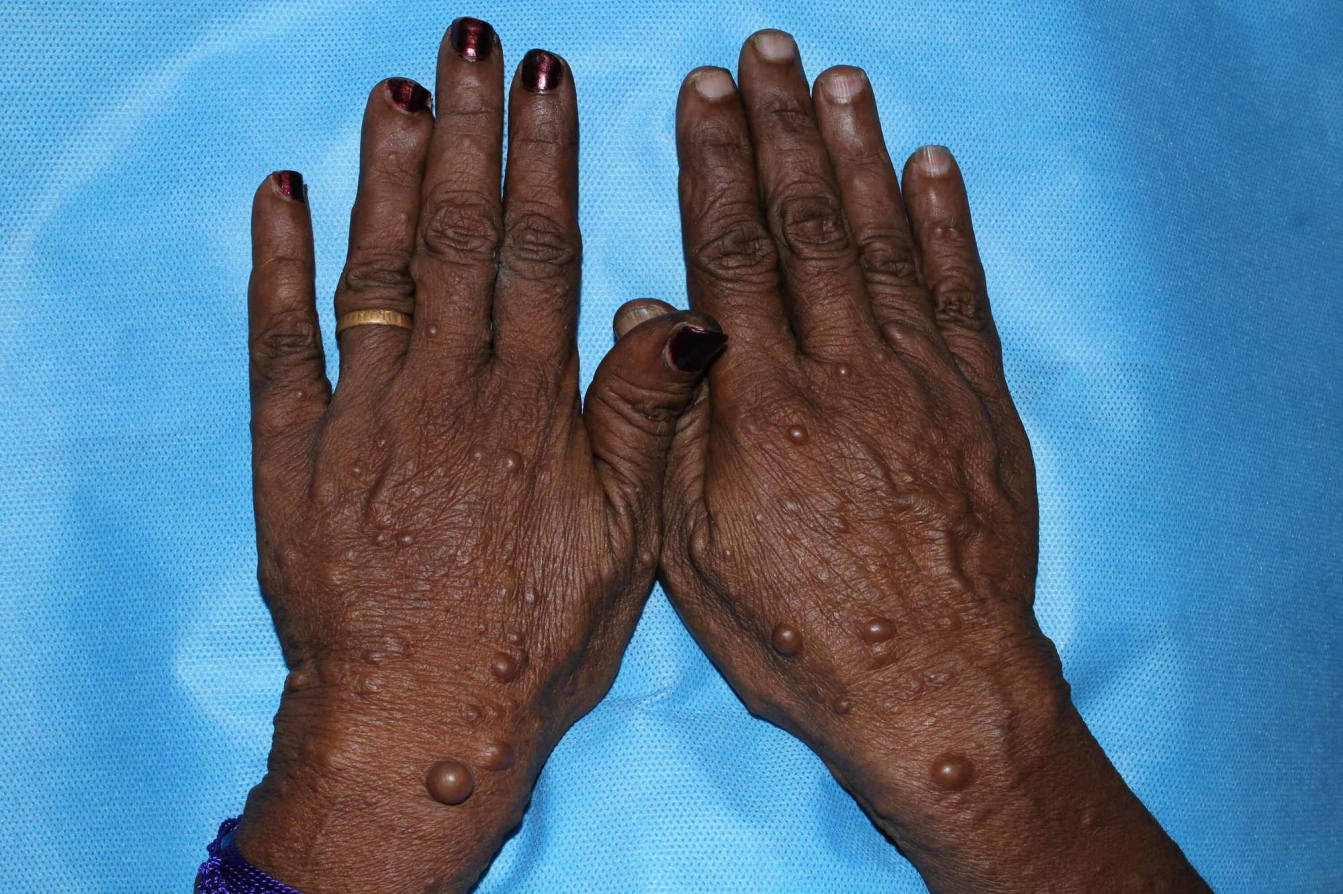
Extra Oral Features : Neurofibramatosis present on the Dorsum of Hands

On enquiring the history, the patient reported that the swelling was present since childhood which had gradually increased in size. There was no associated pain or bleeding associated with the growth of the swelling. The patient also presented with a history of Type I Neurofibromatosis with multiple small tumours present all over the body for which she had previously undergone excision. No pertinent family history was reported [Fig 2].

Intraoral examination revealed a sessile, non-ulcerated, diffuse, unilateral swelling present in the maxillary right area involving the marginal and attached gingiva corresponding to teeth 14-17. The swelling was fibrous, painless and of a non-inflammatory origin. It measured around 17mm in diameter and was firm on palpation. While the probing depth for the teeth was less than 3mm, attachment loss was found to the evident in the teeth associated with the lesion. All the associated teeth were found to be vital, with no decay and no accidental trauma. The radiographs taken in that area did not reveal any alteration. Ultrasound of the swelling revealed it to be of neurologic origin. A provisional diagnosis of NF1 of the gingiva was made and the lesion was planned to be excised in entirety using laser. Prior to excision, we had obtained medical fitness from the Department of Neurology and the Department of Dermatology, SRIHER.

**Figure 3:**
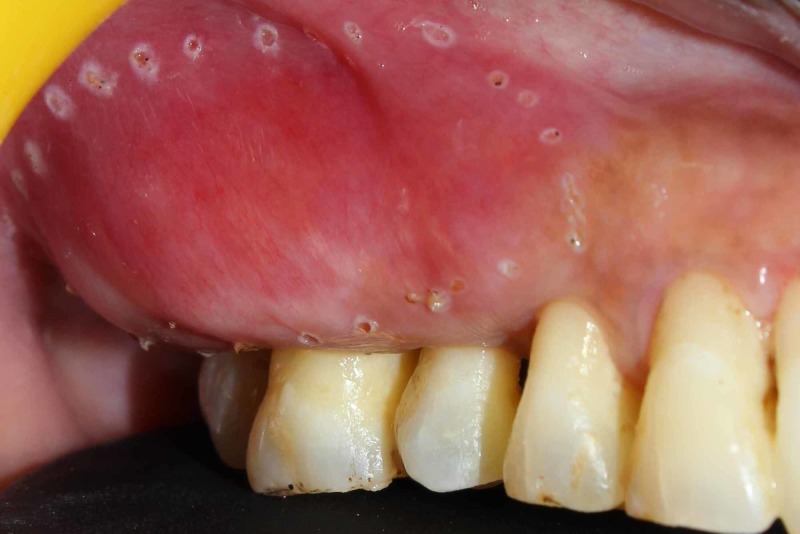
Intra Operative : Lesion outline marked with Diode LASER

**Figure 4:**
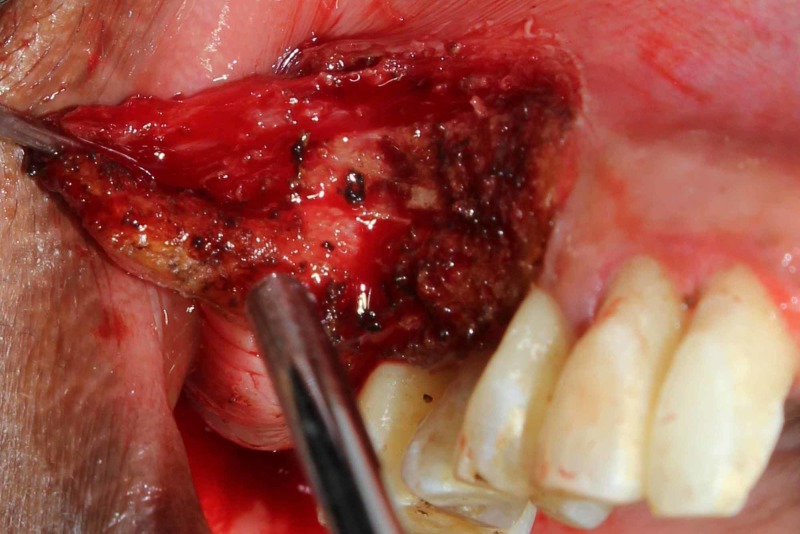
Intra Operative : Excision of the Lesion using Diode LASER

**Figure 5:**
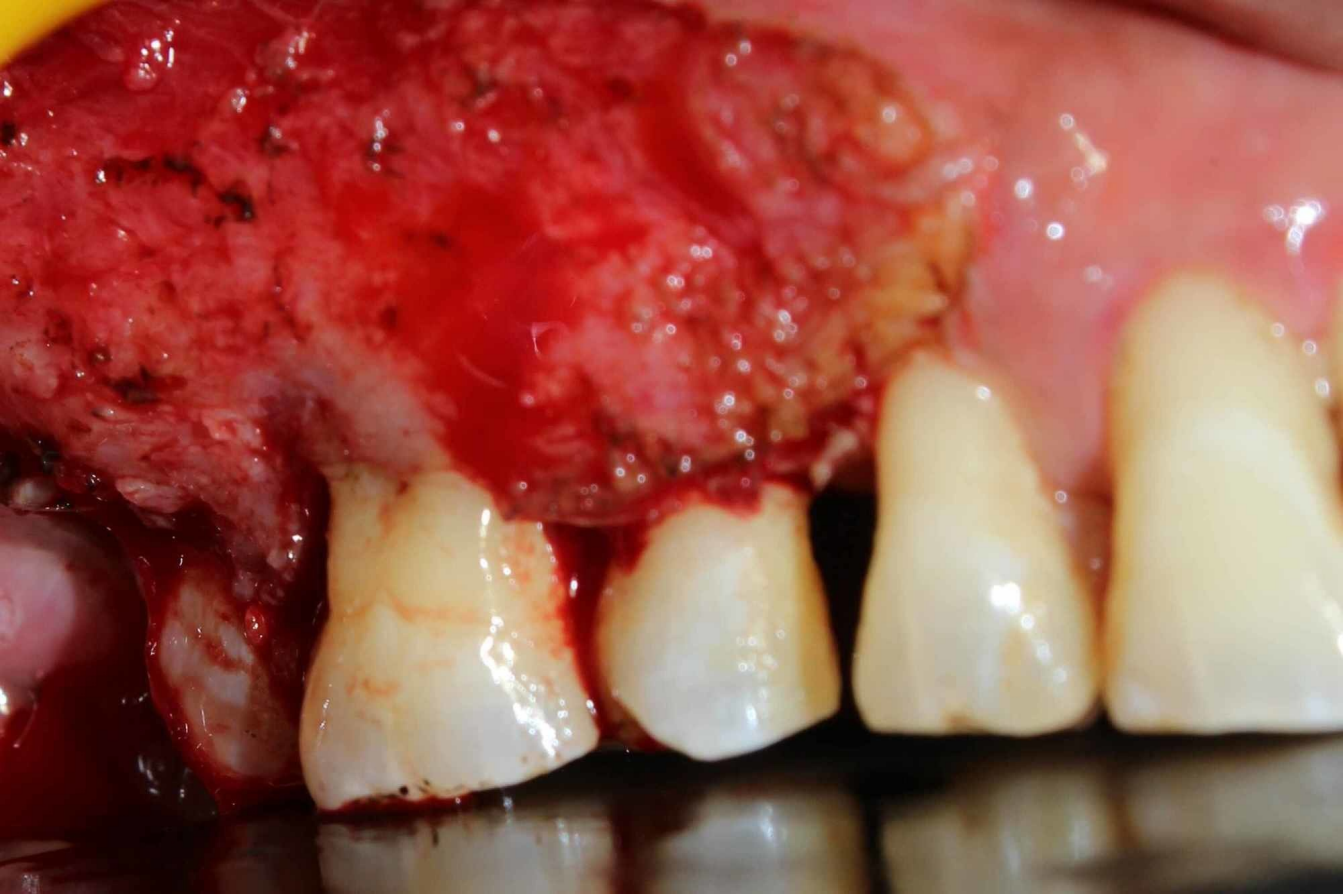
Immediate Post Operative Image

Under local anesthesia, using appropriate laser precautions, the outline of the swelling was marked using laser [Fig 3] and the lesion as a whole was excised using diode laser of 940nm wavelength with 3W power along with 2mm of normal tissue so as to differentiate between the normal and diseased tissue [Fig 4 and Fig 5].

**Figure 6:**
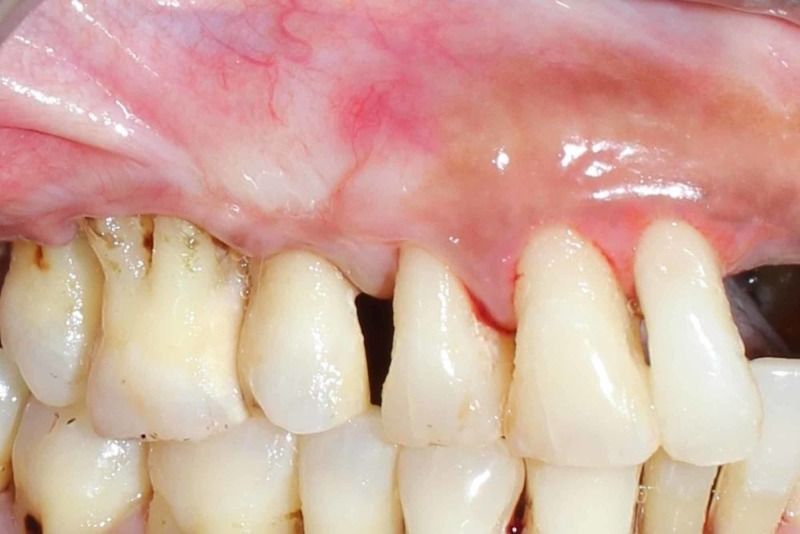
Post Operative : After 6 months healing

Upon achieving hemostasis, the area was thoroughly irrigated with saline and Coe-Pak™ (GC America Inc) surgical dressing was placed. The patient was recalled on day 1 to assess the healing and no complications were observed. The excised tissue was seen histologically which revealed the presence of proliferating spindle cells with wavy nuclei. The underlying connective tissue showed richly cellular nerve tissue comprising of spindle shaped cells with wavy nuclei in collagenous stroma along with few chronic inflammatory cell infiltration and increased vascularity. Review on day 7, three months and six months post-op revealed good healing [Fig 6].

On 6 months post-op, the area of the lesion had healed completely and no indication of recurrence was observed. The patient was referred for further prosthetic management.

## Discussion

Neurofibromatosis type I is an autosomal dominant, multisystem disorder that affects approximately 1 in 3500 people. The earliest historical evidence for NF1 appeared in the 13th century [6]. In 1982, VM Riccardi classified heterogenous neurofibromatosis into eight categories NF I-VIII [7]. NF2 occurs because of mutation in the NF2 gene and is characterized by bilateral vestibular schwannomas and other tumours. NF7 is referred to as Schwannomatosis and is characterized by the presence of multiple schwannomas which is of late-onset and the features of NF1 and NF2 are absent [8]. The gene for NF1 is located on chromosome (17q11.2) and encodes for protein neurofibromin. The gene protein product is a tumour suppressor which is expressed in many cell types primary in neurons, glial cells, Schwann cells and early in melanocyte development. In 1987, Gutmann gave the diagnostic criteria for the diagnosis of NF 1. Out of the seven cardinal criteria, at least two must be present for a person to be diagnosed with neurofibrormatosis [9]. These criteria are:

• Six or more cafe-au-last spots (>5mm pre-puberty, >15mm post-puberty)

• Two or more neurofibromas of any type, or one or more plexiform neurofibromas

• Freckling in the axillae or groin

• Optic glioma

• Two or more Lisch nodules

• Dysplasia of the sphenoid, dysplasia or thinning of long bone cortex

• First degree relative with NF1

The cutaneous manifestations of NF1 include Café-au-lait macules, Axillary/ Inguinal freckling, Neurofibromas and increased base pigmentation. The uncommon findings include the presence of Juvenile Xanthogranuloma, Glomus tumour, Melanoma, Bue-red macules, Pseudoatrophic macules and Nevus anemicus [4]. Histologically, neurofibromatosis exhibits proliferation of delicate spindle cells with thin, wavy nuclei intermingled with neurites in an irregular pattern along with delicate intertwining connective tissue fibrils. The lesional cells are positive for S100 proteins [10]. The management of NF1 is a multidisciplinary approach. Since there is no cure for NF1, therapeutic measures should be aimed towards prevention and control of the complications. The complications of NF1 include malignant transformation (rare), esthetic problems, compromise in function [11]. Surgical management is indicated only if there is a compromise in function of the patient as there is a risk of malignant transformation post surgery. Risk, expected benefits and possible complications should be carefully evaluated prior to surgical intervention.

There have been a number of case reports showing the oral manifestations of NF1 but the presence of NF1 in the gingiva has been rare [12-15]. Shapiro et al. observed the presence of gingival neurofibromatosis in only 5% of all patients with NF1 [16]. The most common finding in the oral cavity in a NF1 patient is the enlargement of the fungiform papillae of the tongue that has been reported in about 50% of the cases. Oral localized neurofibromatosis usually presents as discrete nodules of normal colour and are asymptomatic. The other radiographic features include enlarged mandibular canal, mandibular foramen and mental foramen [17]. The treatment modalities for surgical excision of NF include the conventional scalpel, electrocautery and the usage of diode laser. Solitary lesions are excised depending on the site and extent of the lesion. In our situation, since the lesion was close to 2 cm in diameter, laser excision was preferred to reduce the risk of post-operative bleeding. Electrosurgery was avoided to prevent necrosis of the underlying bone and to prevent any damage to the nerve sheath. Though there was an increase in the surgical time, the beneficial property of diode laser (being the relatively easier ablation of soft tissues along with the hemostatic and bactericidal effects) provided us with better results [18].

## Conclusions

Even though the possibility of finding NF1 in the gingiva is rare, the possibility should not be ruled out. The present case report shows that NF1 can present as a painless, solitary swelling in the gingiva, which when left untreated can result in difficulty in speech, mastication and worsening of the periodontal status of the patient. Current dental treatment for patients affected by neurofibroma should include restoration of true oral health, incorporating comfort function and aesthetics. Newer surgical approaches help in minimizing surgical trauma and improving the quality of life in such patients.
